# Hydrogen Sulfide Prevents Formation of Reactive Oxygen Species through PI3K/Akt Signaling and Limits Ventilator-Induced Lung Injury

**DOI:** 10.1155/2017/3715037

**Published:** 2017-01-31

**Authors:** Sashko Georgiev Spassov, Rosa Donus, Paul Mikael Ihle, Helen Engelstaedter, Alexander Hoetzel, Simone Faller

**Affiliations:** Department of Anesthesiology and Critical Care Medicine, University Medical Center Freiburg, Hugstetter Str. 55, 79106 Freiburg, Germany

## Abstract

The development of ventilator-induced lung injury (VILI) is still a major problem in mechanically ventilated patients. Low dose inhalation of hydrogen sulfide (H_2_S) during mechanical ventilation has been proven to prevent lung damage by limiting inflammatory responses in rodent models. However, the capacity of H_2_S to affect oxidative processes in VILI and its underlying molecular signaling pathways remains elusive. In the present study we show that ventilation with moderate tidal volumes of 12 ml/kg for 6 h led to an excessive formation of reactive oxygen species (ROS) in mice lungs which was prevented by supplemental inhalation of 80 parts per million of H_2_S. In addition, phosphorylation of the signaling protein Akt was induced by H_2_S. In contrast, inhibition of Akt by LY294002 during ventilation reestablished lung damage, neutrophil influx, and proinflammatory cytokine release despite the presence of H_2_S. Moreover, the ability of H_2_S to induce the antioxidant glutathione and to prevent ROS production was reversed in the presence of the Akt inhibitor. Here, we provide the first evidence that H_2_S-mediated Akt activation is a key step in protection against VILI, suggesting that Akt signaling limits not only inflammatory but also detrimental oxidative processes that promote the development of lung injury.

## 1. Introduction

Although mechanical ventilation is a life-saving tool in intensive care medicine, it can culminate in severe lung injury, even in the previously healthy lung [1, 2]. This ventilator-associated lung injury arises from constant cyclical stretching and is characterized by vast pathohistological tissue damage, for example, alveolar wall disruption, edema formation, influx of immune competent cells, release of proinflammatory cytokines, and the excessive formation of reactive oxygen species (ROS) [3–5]. These detrimental events may generate a vicious cycle, leading to systemic inflammation and multiorgan failure. The introduction of low tidal volume ventilation has improved clinical outcome [6]; however, morbidity and mortality still remain unacceptably high and no therapeutic option is available at present.

Hydrogen sulfide (H_2_S), for long only recognized as a toxic agent, has been identified as an endogenously produced gaseous transmitter, mediating, for instance, vasodilation or regulation of inflammatory responses [7]. Exogenous application of H_2_S, either as an inhaled gas or as a soluble H_2_S-releasing compound, revealed its anti-inflammatory, antiapoptotic, and antioxidative properties in a series of organ injury models [7].

We and others have recently shown that H_2_S also protects from experimental ventilator-induced lung injury (VILI) in rodents [8–11]. Here, H_2_S provided lung protection by limiting proinflammatory cytokine release and neutrophil transmigration [8–11] and enhancing antioxidative responses [10]. However, despite the identification of H_2_S-induced activation of genes involved in prevention of apoptosis and inflammation [11] or antioxidation [10], very little is known about the molecular signaling pathways mediating these effects. In addition, the functional role of H_2_S in prevention of oxidative responses in VILI remains unknown. Although the production of ROS reflects a key step in the development of lung injury during mechanical ventilation, it has not yet been examined in the light of H_2_S. In the present study, we provide the first evidence that inhaled H_2_S prevents ROS formation in VILI. Moreover, we found that H_2_S inhalation induced the activation of Akt. Akt phosphorylation plays a functional role in VILI [12–16] and mediates anti-inflammatory and antioxidative signaling [12, 17], but this had not yet been studied in the light of H_2_S during mechanical ventilation. Moreover, we demonstrate for the first time that inhibition of Akt signaling in H_2_S treated and ventilated mice reestablishes both ROS production and lung injury, indicating that H_2_S affects antioxidative signaling by activating Akt signaling, which may subsequently prevent ROS formation, while protecting against VILI.

## 2. Materials and Methods

### 2.1. Animals

All animal experiments were performed in accordance with the guidelines of the local animal care commission (University of Freiburg, Ethics Committee and Regierungspräsidium Freiburg, Freiburg, Germany, Permission number G-07/25 and number G-12/73) and in conformance with the journals' requirements for human and animal trials. All possible efforts were undertaken to avoid animal suffering at each stage of the experiments.

### 2.2. Experimental Setup

Male C57BL/6N mice, weighing 24–26 g, were obtained from Charles River Laboratories (Sulzfeld, Germany). Preparation and ventilation were performed as described earlier in detail [9, 12]. In brief, mice were anesthetized intraperitoneally (*i.p.*; 90 mg/kg ketamine and 0.9 mg/kg acepromazine). An arterial line and a tracheal tube were inserted. In the case of mechanical ventilation, mice were connected to a rodent ventilator (Voltek Enterprises, Toronto, ON, Canada) set to a tidal volume of 12 ml/kg, frequency of 80–90 breaths/minute, and positive end-expiratory pressure of 2 cmH_2_O for 6 hours. Directly after onset of mechanical ventilation, muscular relaxation was induced (2 mg/kg,* i.p.*) and maintained as needed by injection of pancuronium. Likewise, general anesthesia was maintained by continuous* i.p.* administration of ketamine and acepromazine as needed. The animal experiments were run in two independent sets. In each experimental set, mice were randomized into 4 groups with *n* = 6/group.

*Set 1*. Two control groups were allowed to spontaneously breathe either synthetic air (air control) or synthetic air supplemented with 80 parts per million (ppm) of H_2_S (H_2_S control) for 6 h. Another two groups were ventilated with synthetic air (air vent) or synthetic air with 80 ppm H_2_S (H_2_S vent) for 6 h.

*Set 2*. Mice of all groups were treated* i.p.* with either the phosphoinositide 3-kinase (PI3K)/Akt inhibitor LY294002 (LY; 5 *µ*g/g, solved in 0.9% saline solution containing 2% dimethyl sulfoxide) or its solvent (vehicle; 0.9% saline solution containing 2% dimethyl sulfoxide) 1 h before the onset of mechanical ventilation or control preparation [12, 14]. Unventilated mice were injected with LY and allowed to breathe synthetic air spontaneously for 6 h (control + LY). Ventilated mice were treated with vehicle and received either synthetic air (air vent) or synthetic air supplemented with 80 ppm H_2_S (H_2_S vent). The last group received LY and was ventilated with synthetic air supplemented with 80 ppm H_2_S (H_2_S vent + LY).

In addition, mice were either treated as controls or ventilated as indicated in the absence or presence of LY294002 in order to analyze Akt phosphorylation in the lungs by Western blot.

### 2.3. Tissue and Fluid Sampling

At the end of each experiment, mice were sacrificed by an overdosed* i.p.* injection of ketamine (180 mg/kg) and acepromazine (1.8 mg/kg). Tissue and fluid sampling procedures were always done in the same chronological order within 15 min to minimize postmortem interval artefacts. Bronchoalveolar lavage (BAL) fluid was gained by carefully flushing the right lung lobes. The left lungs were preserved for Western blot analysis and histological examination as previously described [9, 12].

### 2.4. Neutrophil Analysis

BAL fluid cells were separated by cellspin® (Tharmac GmbH, Waldsolms, Germany) and the fraction of neutrophils was determined by direct microscopic count after Diff-Quick® staining (Siemens Healthcare Diagnostics, Eschborn, Germany) [9, 12].

### 2.5. Cytokine Measurements

BAL supernatant aliquots were analyzed using a macrophage inflammatory protein-1*β* (MIP-1*β*) ELISA kit (R&D Systems GmbH, Wiesbaden, Germany) according to the manufacturers' instructions.

### 2.6. Histological Analysis

Cryosections (6 *µ*m) of the left lung were stained with hematoxylin and eosin (HE) and analyzed as described earlier [9, 12]. The degree of lung damage was determined by a VILI-score as previously described [9, 12].

### 2.7. Detection of Oxidative Stress (RONS)

Cryosections of unfixed frozen lung tissue samples were stained with dihydroethidium (DHE, Life Technologies GmbH, Darmstadt, Germany) and analyzed as described earlier [18] in order to detect reactive oxygen and nitrogen species (RONS). Densitometric analyses were performed with Zen software (blue edition, Carl Zeiss) and results are depicted as fold induction referring to the untreated control group.

### 2.8. Detection of Glutathione

With this method, reduced thiol is directly stained in the cell. Tissue sections were prepared as described above. ThiolTrackerTM Violet (Life Technologies) was used to detect glutathione in lung tissue as previously described [19]. Densitometric analyses were performed with Zen software (blue edition, Carl Zeiss) and results are shown as fold induction in comparison to the untreated control group.

### 2.9. Immunoblotting

Western blotting was performed as described recently [12]. Membranes were incubated with antibodies against NADPH oxidases 1 and 4 (Nox1; Nox4; Santa Cruz Biotechnology Inc., Heidelberg, Germany), Nox2 (Becton Dickinson GmbH, Heidelberg, Germany), phosphorylated Akt, or Akt (Cell Signaling, Leiden, The Netherlands). Normalization in order to control equal protein loading was performed by stripping and reblotting of the membranes with glyceraldehyde-3-phosphate-dehydrogenase (GAPDH; Enzo Life Sciences GmbH, Lörrach, Germany). Data represent fold induction of indicated proteins with respect to GAPDH or total Akt after densitometric analysis (Fusion FX7, Peqlab, Germany) as indicated.

### 2.10. Statistical Analysis

For the two experimental settings, mice were randomized into groups of *n* = 6 each. Prior to the study, power calculations were performed in order to define group sizes. Some data points had to be excluded because of technical problems. Thus subsequent lung analyses were done with *n* = 4–6 samples per group as indicated. Graphs represent means ± standard error of means (SEM) and were created with SigmaPlot 11.0 software (Systat Software Inc., Erkrath, Germany). Data were further analyzed for normal variation prior to one way analysis of variance (ANOVA) followed by Tukey's post hoc test. *P* < 0.05 was considered significant. All calculations were performed with GraphPad Prism 5 (GraphPad Software, Inc., La Jolla, CA, USA).

## 3. Results

### 3.1. Effect of H_2_S on Ventilator-Induced Lung Damage

**Figure 1:**
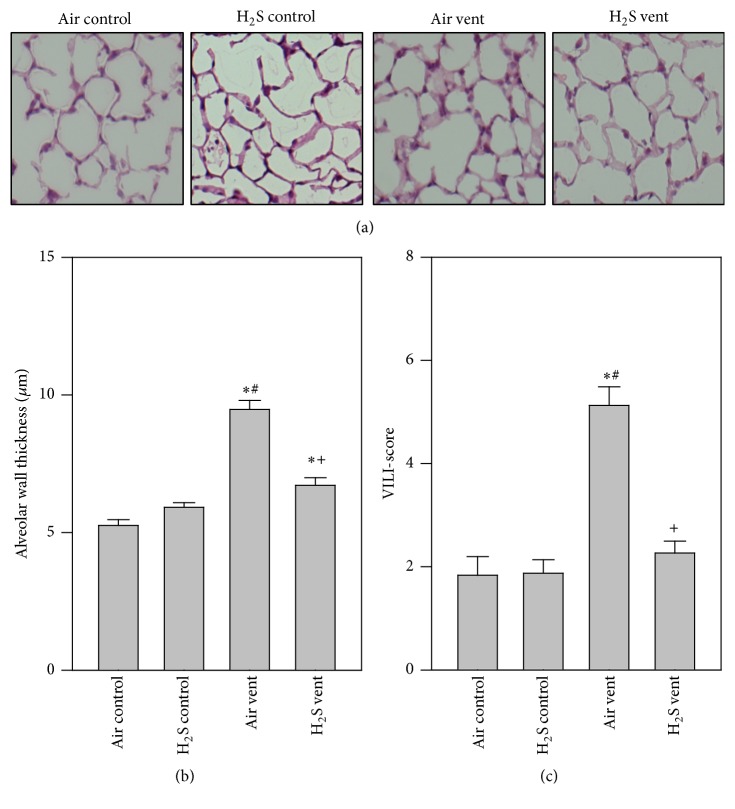
Effect of H_2_S on ventilator-induced lung damage. Mice spontaneously breathed air (air control) or air supplemented with 80 ppm H_2_S (H_2_S control) for 6 h, or they were mechanically ventilated with 12 ml/kg for 6 h with either air alone (air vent) or air supplemented with 80 ppm H_2_S (H_2_S vent). Lung tissue sections were stained with hematoxylin and eosin. Representative pictures are shown for each experimental group as indicated (a). High power fields were randomly assigned to measure alveolar wall thickness (b) and to calculate a ventilator-induced lung injury- (VILI-) score (c). Data represent means ± SEM for *n* = 6/group. ANOVA (Tukey's post hoc test), ^*∗*^*P* < 0.05 versus air control group; ^#^*P* < 0.05 versus H_2_S control group; ^+^*P* < 0.05 versus air vent group.

Alveolar wall thickness and VILI-score were measured in order to determine the histopathological level of lung damage as a consequence of mechanical ventilation. Compared to air or H_2_S treated controls, thicknesses of alveolar walls were significantly increased in air ventilated animals. In contrast, alveolar wall thickness of H_2_S ventilated mice was reduced, almost back to control levels (Figures 1(a) and 1(b)). Analysis of an overall VILI-score yielded the same results (Figure 1(c)).

### 3.2. Effect of H_2_S on Inflammation in VILI

**Figure 2:**
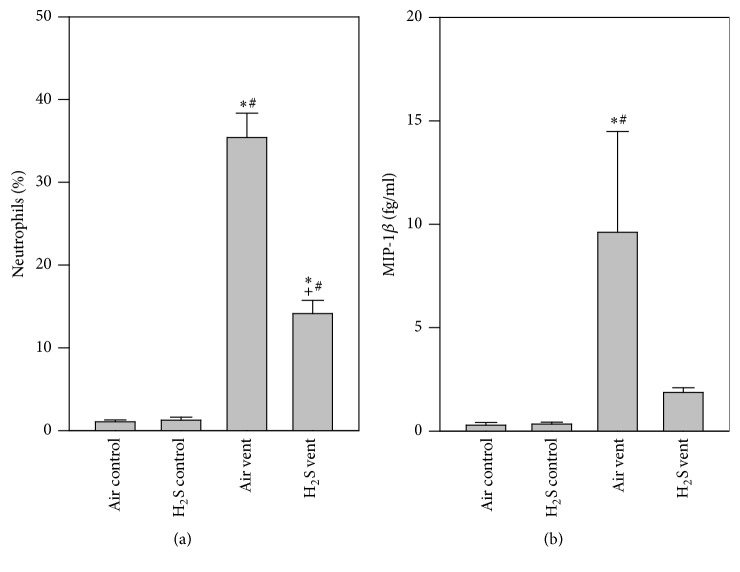
Effect of H_2_S on inflammation in VILI. Mice spontaneously breathed air (air control) or air supplemented with 80 ppm H_2_S (H_2_S control) for 6 h, or they were mechanically ventilated with 12 ml/kg for 6 h with either air alone (air vent) or air supplemented with 80 ppm H_2_S (H_2_S vent). Bronchoalveolar lavage was performed in the right lung. The relative amount of neutrophils (a) was determined by cytospin analysis and the content of MIP-1*β* (b) was determined by ELISA. Data represent means ± SEM for *n* = 6/group. ANOVA (Tukey's post hoc test), ^*∗*^*P* < 0.05 versus air control group; ^#^*P* < 0.05 versus H_2_S control group; ^+^*P* < 0.05 versus air vent group.

While neutrophils were almost absent in both groups of nonventilated animals, mechanical ventilation with air led to a significant influx of these cells. In sharp contrast, application of H_2_S during ventilation reduced neutrophil counts (Figure 2(a)). Compared to air or H_2_S controls, MIP-1*β* release into the bronchoalveolar space was elevated by air ventilation and was prevented in the presence of H_2_S (Figure 2(b)).

### 3.3. Effect of H_2_S on Oxidative Stress in VILI

**Figure 3:**
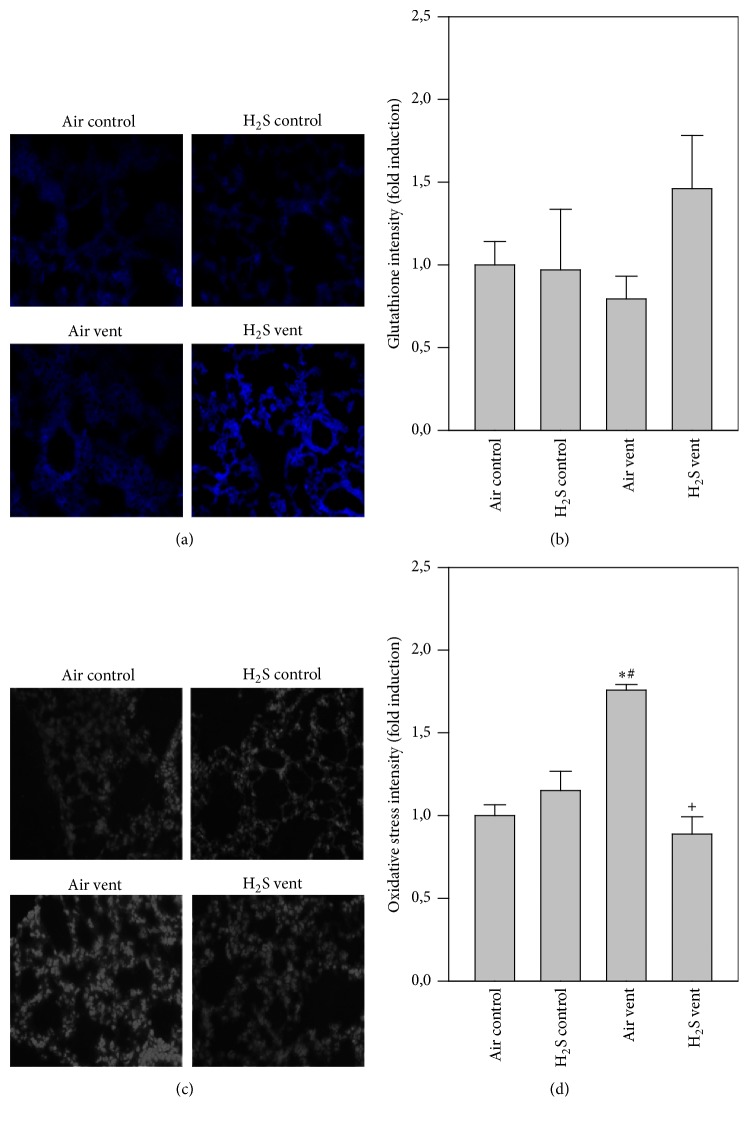
Effect of H_2_S on oxidative stress in VILI. Mice spontaneously breathed air (air control) or air supplemented with 80 ppm H_2_S (H_2_S control) for 6 h, or they were mechanically ventilated with 12 ml/kg for 6 h with either air alone (air vent) or air supplemented with 80 ppm H_2_S (H_2_S vent). Lung tissue sections were stained for glutathione (a) or RONS (oxidative stress, (c)). Representative pictures are shown for each experimental group as indicated (a + c). Glutathione and RONS fluorescence intensity was measured and expressed as fold induction compared to air control group (b + d). Data represent means ± SEM for *n* = 4/group. ANOVA (Tukey's post hoc test), ^*∗*^*P* < 0.05 versus air control group; ^#^*P* < 0.05 versus H_2_S control group; ^+^*P* < 0.05 versus air vent group.

While antioxidant glutathione levels were alike in H_2_S controls and air ventilated mice compared to nonventilated controls, glutathione levels were enhanced by H_2_S application during mechanical ventilation (blue staining, Figure 3(a)). This result was confirmed by quantitative analysis of all groups (Figure 3(b)). Compared to nonventilated controls, air ventilation clearly increased RONS formation in the lungs (grey staining, Figure 3(c)). H_2_S treatment during ventilation completely prevented RONS production that was as low as control levels (Figures 3(c) and 3(d)).

### 3.4. Effect of H_2_S on Nox and Akt Protein Expression in VILI

**Figure 4:**
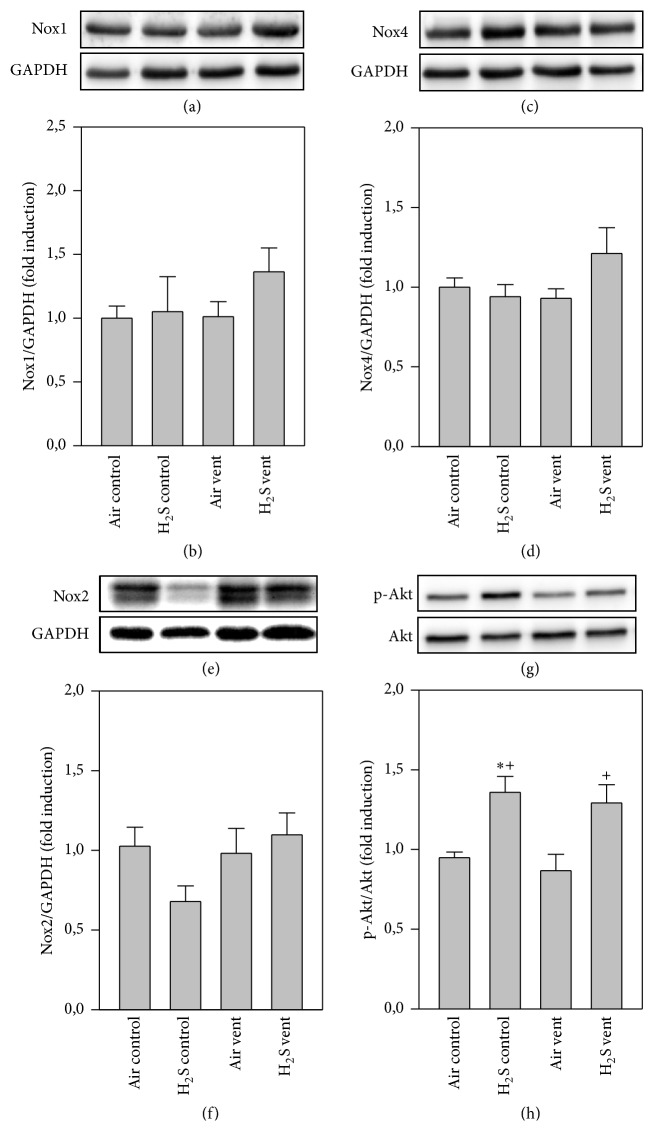
Effect of H_2_S on Nox and Akt protein expression in VILI. Mice spontaneously breathed air (air control) or air supplemented with 80 ppm H_2_S (H_2_S control) for 6 h, or they were mechanically ventilated with 12 ml/kg for 6 h with either air alone (air vent) or air supplemented with 80 ppm H_2_S (H_2_S vent). Western blot analysis was performed with lung tissue homogenates. Representative Western blots are shown for Nox1 (a), Nox4 (c), Nox2 (e), p-Akt (g), and the corresponding housekeeping protein (lower panel in (a), (c), (e), and (g)). Densitometric analyses of all samples were normalized to GAPDH or total Akt and expressed as fold induction for Nox1 (b), Nox4 (d), Nox2 (f), and p-Akt (h). Data represent means ± SEM for *n* = 4 (H_2_S control group) or *n* = 6/group. ANOVA (Tukey's post hoc test), ^*∗*^*P* < 0.05 versus air control group; ^+^*P* < 0.05 versus air vent group.

Since ROS are mainly produced by NADPH oxidases [20], expressions of Nox1, Nox2, and Nox4 proteins were determined in the lungs by Western blot analysis. There were no differences between groups for Nox1 (Figures 4(a) and 4(b)) and Nox4 (Figures 4(c) and 4(d)), but a trend towards decreased Nox2 expression in the H_2_S control group compared to all other groups (Figures 4(e) and 4(f)). Akt represents another key molecule in inflammatory and oxidative signaling [17]. In sharp contrast to nonventilated air controls or air ventilated mice, expression of phosphorylated Akt was clearly upregulated in H_2_S treated groups irrespective of whether animals were ventilated or not (Figures 4(g) and 4(h)).

### 3.5. Effect of Akt Inhibition and H_2_S on Nox and Akt Protein Expression

**Figure 5:**
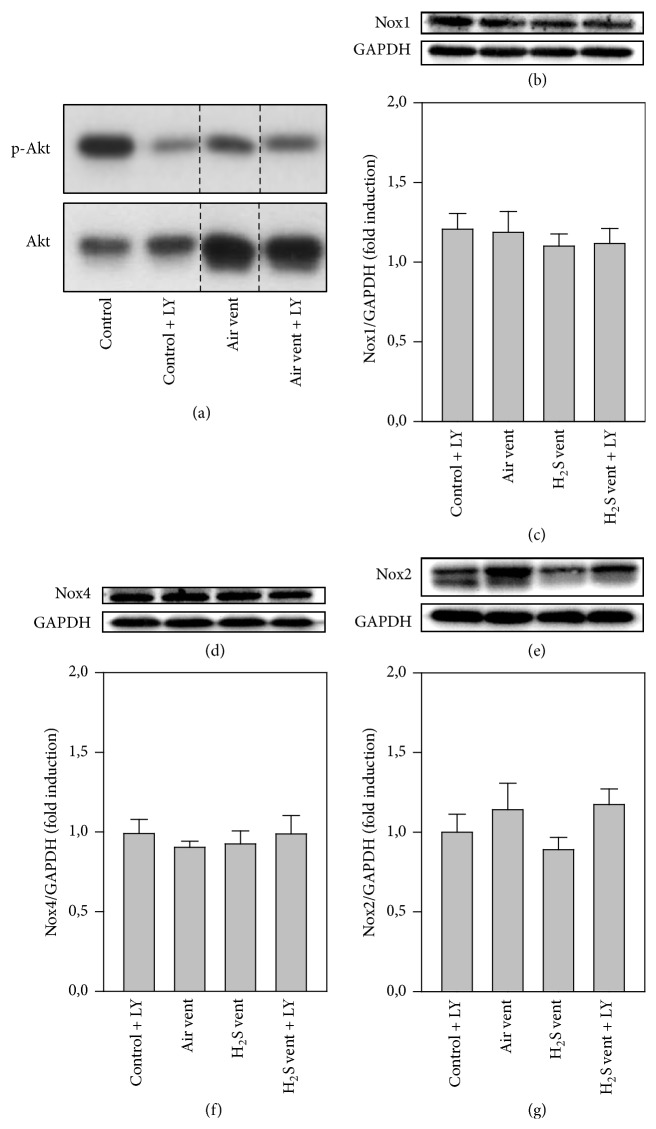
Effect of Akt inhibition and H_2_S on Nox and Akt protein expression. (a) Control mice were treated without (control) or with LY294002 (control + LY) and spontaneously breathed air for 6 h. Ventilated mice (12 ml/kg, 6 h) were treated without (air vent) or with LY294002 (air vent + LY). Western blot analysis was performed with lung tissue homogenates for p-Akt (upper lane) and total Akt (lower lane). (b–g) Control mice were treated with LY294002 and spontaneously breathed air (air control + LY) for 6 h. Ventilated mice (12 ml/kg, 6 h) were treated with either vehicle and inhaled air (air vent) or air supplemented with 80 ppm H_2_S (H_2_S vent), or they received LY294002 and inhaled air supplemented with 80 ppm H_2_S (H_2_S vent + LY). Western blot analysis was performed with lung tissue homogenates. Representative Western blots are shown for Nox1 (b), Nox4 (d), Nox2 (f), and the corresponding housekeeping protein (lower panel in (c), (e), and (g)). Densitometric analyses of all samples were normalized to GAPDH and expressed as fold induction for Nox1 (c), Nox4 (e), and Nox2 (g). Data represent means ± SEM for *n* = 6/group. ANOVA.

In order to determine the functional relevance of Akt for the protective effects of H_2_S against VILI, in a second set of experiments, animals were treated with the phosphoinositide 3-kinase (PI3K)/Akt inhibitor LY294002. After 6 h, Akt phosphorylation was clearly reduced by inhibition of PI3K/Akt due to LY294002 treatment in both control and ventilated mice (Figure 5(a)). Nox1 (Figures 5(b) and 5(c)) and Nox4 (Figures 5(d) and 5(e)) protein expression was not different between groups after Akt inhibition. Nox2 expression was slightly reduced in H_2_S ventilated mice compared to air ventilation or control treatment, while this reduction was again abrogated under Akt inhibition during H_2_S ventilation (Figures 5(f) and 5(g)).

### 3.6. Effect of Akt Inhibition on H_2_S-Mediated Effects in Oxidative Stress

**Figure 6:**
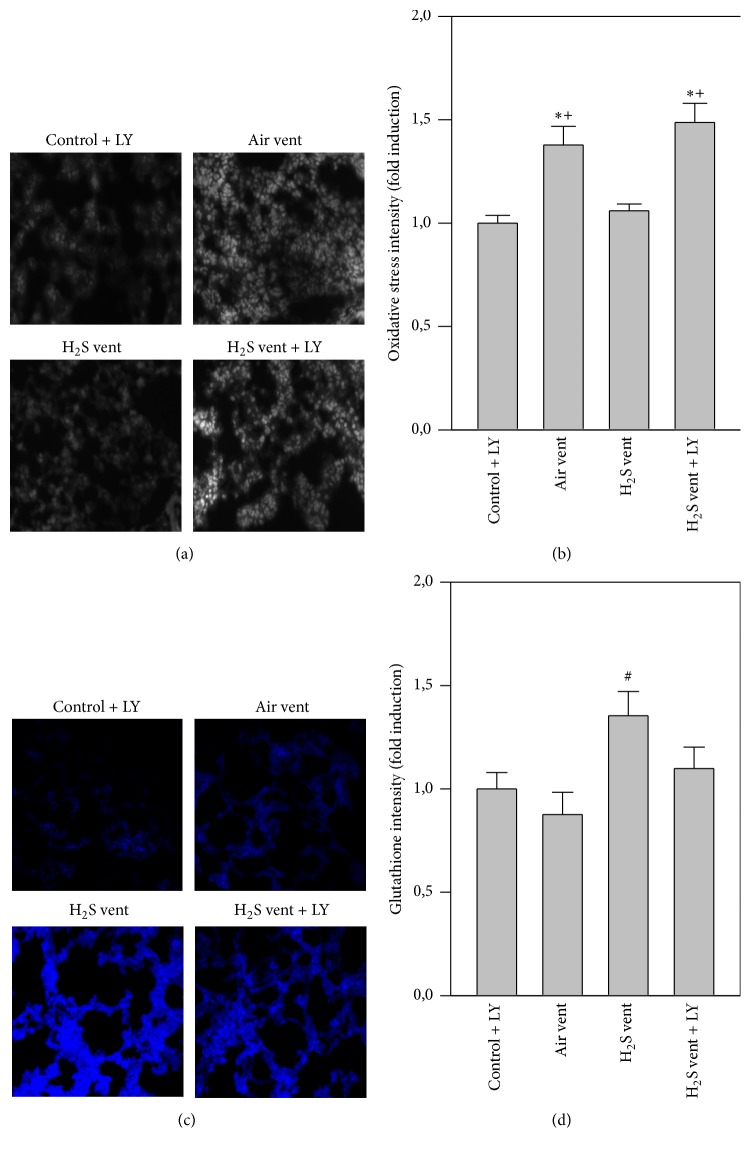
Effect of Akt inhibition on H_2_S-mediated effects in oxidative stress. Control mice were treated with LY294002 and spontaneously breathed air (air control + LY) for 6 h. Ventilated mice (12 ml/kg, 6 h) were treated with either vehicle and inhaled air (air vent) or air supplemented with 80 ppm H_2_S (H_2_S vent), or they received LY294002 and inhaled air supplemented with 80 ppm H_2_S (H_2_S vent + LY). Lung tissue sections were stained for RONS (oxidative stress, (a)) or glutathione (c). Representative pictures are shown for each experimental group as indicated (a + c). RONS and glutathione fluorescence intensity was measured and expressed as fold induction compared to air control group (b + d). Data represent means ± SEM for *n* = 5/group. ANOVA (Tukey's post hoc test), ^*∗*^*P* < 0.05 versus air control + LY group; ^#^*P* < 0.05 versus air vent group; ^+^*P* < 0.05 versus H_2_S vent group.

Air ventilation alone promoted RONS formation in the lungs compared to control + LY treated mice. H_2_S ventilation reduced RONS production back to control levels, while additional inhibition of Akt again augmented RONS formation despite the presence of H_2_S (Figures 6(a) and 6(b)). Compared to nonventilated control + LY and air ventilated lungs ventilation with H_2_S enhanced antioxidant glutathione formation (blue stain, Figures 6(c) and 6(d)). In contrast, Akt inhibition during H_2_S ventilation prevented glutathione synthesis (Figures 6(c) and 6(d)).

### 3.7. Effect of Akt Inhibition on H_2_S-Mediated Effects in Inflammation

**Figure 7:**
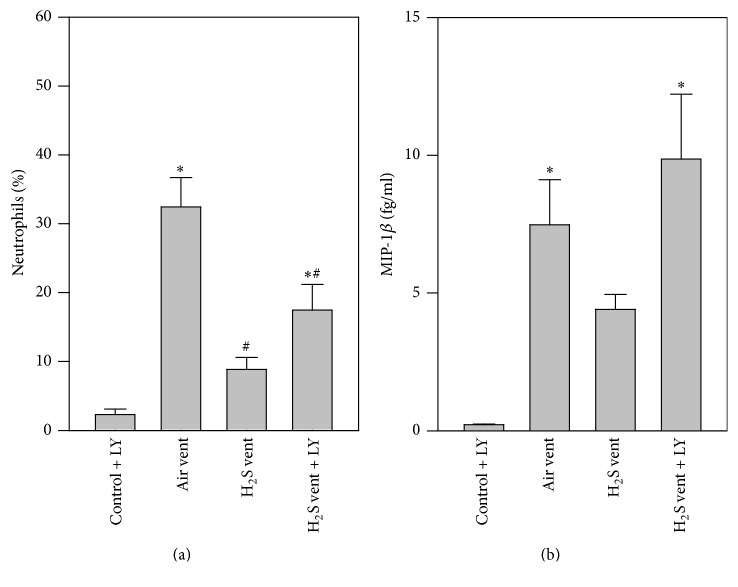
Effect of Akt inhibition on H_2_S-mediated effects in inflammation. Control mice were treated with LY294002 and spontaneously breathed air (control + LY) for 6 h. Ventilated mice (12 ml/kg, 6 h) were treated with either vehicle and inhaled air (air vent) or air supplemented with 80 ppm H_2_S (H_2_S vent), or they received LY294002 and inhaled air supplemented with 80 ppm H_2_S (H_2_S vent + LY). Bronchoalveolar lavage was performed in the right lung. The relative amount of neutrophils (a) was determined by cytospin analysis and the content of MIP-1*β* (b) was determined by ELISA. Data represent means ± SEM for *n* = 6/group. ANOVA (Tukey's post hoc test), ^*∗*^*P* < 0.05 versus air control + LY group; ^#^*P* < 0.05 versus air vent group.

Next, the effect of Akt inhibition on the inflammatory response during H_2_S ventilation was uncovered. While neutrophil counts were low in the control + LY group, air ventilation alone clearly augmented neutrophil sequestration into the bronchoalveolar space. H_2_S ventilation resulted in a reduction in neutrophils. As a consequence of Akt inhibition during H_2_S ventilation, neutrophil counts again increased (Figure 7(a)). Similar results were obtained with respect to MIP-1*β*. Compared to control + LY both air and H_2_S + LY ventilation augmented MIP-1*β* release into the alveolar space, while H_2_S alone inhibited MIP-1*β* (Figure 7(b)).

### 3.8. Effect of Akt Inhibition on H_2_S-Mediated Effects in Ventilator-Induced Lung Damage

**Figure 8:**
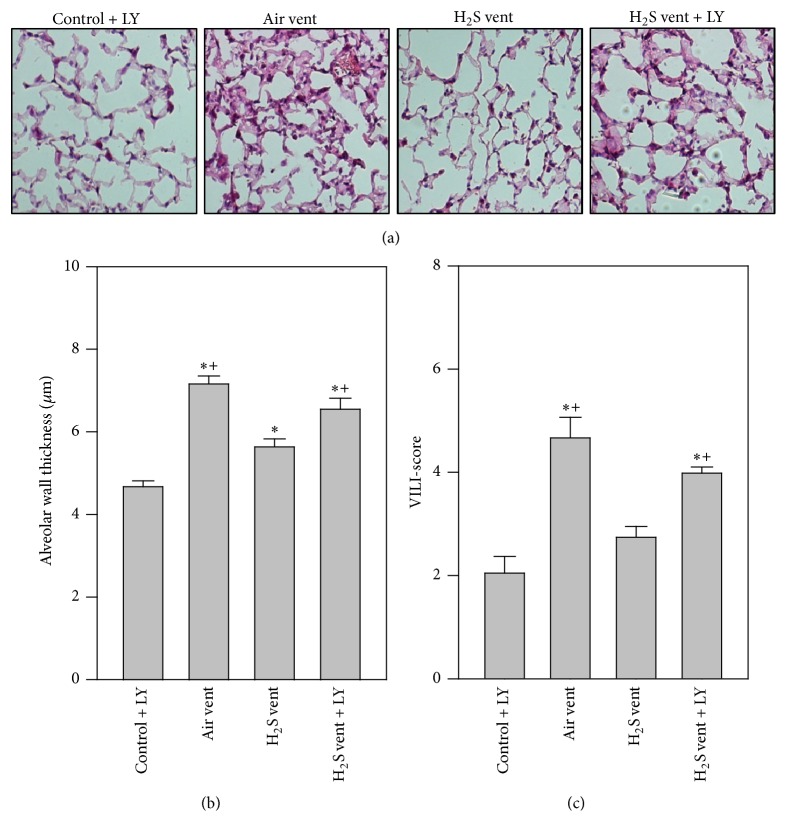
Effect of Akt inhibition on H_2_S-mediated effects in ventilator-induced lung damage. Control mice were treated with LY294002 and spontaneously breathed air (control + LY) for 6 h. Ventilated mice (12 ml/kg, 6 h) were treated with either vehicle and inhaled air (air vent) or air supplemented with 80 ppm H_2_S (H_2_S vent), or they received LY294002 and inhaled air supplemented with 80 ppm H_2_S (H_2_S vent + LY). Lung tissue sections were stained with hematoxylin and eosin. Representative pictures are shown for each experimental group as indicated (a). High power fields were randomly assigned to measure alveolar wall thickness (b) and to calculate a ventilator-induced lung injury- (VILI-) score (c). Data represent means ± SEM for *n* = 6/group. ANOVA (Tukey's post hoc test), ^*∗*^*P* < 0.05 versus control + LY group; ^+^*P* < 0.05 versus H_2_S vent group.

Finally, lung injury parameters under Akt inhibition were determined. Here, alveolar wall thickening was clearly enhanced by air ventilation compared to control + LY. H_2_S ventilation alone reduced alveolar wall thickness which was reversed by additional Akt inhibition (Figures 8(a) and 8(b)). Likewise, the elevated VILI-score caused by mechanical ventilation was prevented by H_2_S inhalation. This effect was completely reversed when Akt was inhibited (Figure 8(c)).

## 4. Discussion

The beneficial effect of H_2_S application in protection from lung injury has been shown in a series of animal models [7]. In VILI, the reduction in inflammatory mediators, edema formation, and lung tissue disruption can be achieved by administration of exogenous H_2_S under experimental conditions [8–11]. In order to further develop the understanding of H_2_S protective effects and to evaluate a potential clinical use in the future, the molecular signaling pathways involved warrant investigation.

In the present study, we demonstrate that moderate tidal volume ventilation with 12 ml/kg for 6 h evokes substantial lung injury in mice as seen by edema formation or cellular influx. This is in line with previous reports by us and by others [8–11]. Adding 80 ppm H_2_S to inhaled air markedly limits the injurious response to mechanical ventilation and elicits lung protection [8, 9, 11]. However, we did not measure H_2_S levels in fluids or tissues. Therefore, to exclude detrimental effects of a potentially high concentration of 80 ppm H_2_S alone, we included a group of mice, spontaneously breathing H_2_S for 6 h. Our results are in line with previous studies, indicating no acute or chronic changes in mice after short or long term exposure to 80 ppm H_2_S [21, 22]. These beneficial effects have also been confirmed in other lung injury models, for example, hyperoxia-induced lung injury [18], pulmonary inflammation [23], or particulate matter-induced lung injury [24]. In all of these models, it was assumed that lung protection can be achieved mainly by inhibition of inflammatory processes. Our current data support these findings. H_2_S inhalation during mechanical ventilation reduced both neutrophil influx and the release of the proinflammatory cytokine MIP-1*β* into the alveolar space. Both are known to directly contribute to the development of VILI [25]. Moreover, recent studies have shown limitation of adhesion and rolling in leukocytes [26, 27], also suggesting an impact of H_2_S on neutrophils in the current study. Nonetheless, the underlying molecular signaling pathways responsible for limiting this inflammatory response in VILI by H_2_S treatment are widely unknown. First attempts in this direction have recently been published [11]. For instance, inhibition of activating transcription factor 3 signaling partially prevented the anti-inflammatory and protective effects of H_2_S in VILI [11], giving the first insights into H_2_S molecular signaling. However, since activating transcription factor 3 signaling was not completely responsible for the observed protection from VILI by H_2_S, it appears likely that other molecular signaling pathways are also orchestrated by H_2_S in the present model.

H_2_S has also been shown to act against oxidative responses thereby limiting organ and cell injury [7]. In in vivo models of left ventricular remodeling in smoking rats [28], or in in vitro models of neuronal oxidative glutamate injury [29, 30], basal levels of antioxidative acting glutathione increased upon H_2_S treatment. Moreover, H_2_S administration during high tidal volume ventilation raised total glutathione and induced several antioxidative genes [10, 11]. Our data add to these findings by showing elevated glutathione levels in the ventilated and H_2_S treated group, suggesting that H_2_S also induces antioxidative signaling pathways in VILI.

Nonetheless, the regulation and functional relevance of oxidative signaling in H_2_S mediated protection from VILI remain elusive at present. Irrespective of the underlying insult, the excessive production of ROS in acute lung injury exacerbates lung damage and inflammatory responses [31]. With respect to mechanical ventilation, it is well known that constant cyclical stretching stimulates ROS production in alveolar epithelial cells [4] and further promotes ROS formation from transmigrated inflammatory cells [32]. In the present model, we therefore first proved that moderate tidal volume ventilation induces oxidative stress. Similar to more recent reports in rodent VILI models, we observed a significant increase of RONS upon mechanical ventilation [19, 33–35]. In contrast, supplemental H_2_S resulted in a complete prevention of RONS formation during ventilation. The obtained results in our VILI-model are in line with findings from other lung injury models. For instance, it has been previously shown that application of H_2_S can decrease ROS formation in hyperoxia-induced lung injury [18], particulate matter-induced lung injury [24], or a lung transplantation model [36]. In all of these studies, the H_2_S-mediated reduction of oxidative stress was associated with attenuation of lung damage and inflammation. The observed antioxidative effects of H_2_S treatment in our setting may in part be explained by the potential of the gas to scavenge free radicals [7], or by upregulation of glutathione persulfide production in mitochondria [37]. In addition, it appears very likely that the process of reactive oxygen and/or nitrogen species formation involves molecular signaling pathways. It is important to note that especially ROS are largely generated by NADPH oxidases (Nox) [38] and that Nox1, Nox2, and Nox4 are predominantly expressed in the lung [31]. Moreover, previous studies showed Nox induction by cyclic strain [4] and their involvement in lung injury has been postulated [39–41]. Against this background, we examined the role of H_2_S on Nox expression in our model. We could not detect a significant difference in protein expression of any Nox enzyme between air ventilated and H_2_S ventilated mice. However, we detected a trend towards reduced Nox2 expression in mice, spontaneously breathing H_2_S for 6 h, indicating at least a minor effect of H_2_S on Nox expression in the current model. These results were surprising and it remains unclear whether our findings may be owed to great individual variances, failure in detection in whole lung homogenates, or the time point of analysis. It might be possible that Nox expression peaks earlier or later than after 6 h of H_2_S treatment. We therefore decided to additionally examine signaling molecules further upstream of ROS and Nox.

For instance, PI3K/Akt signaling has been described to activate Nox, subsequently producing ROS [20, 42]. In addition, results from more recent studies demonstrated PI3K/Akt involvement in anti-inflammatory and antioxidative signaling [12, 17] as well as being of functional relevance in VILI [12–16]. However, both increases [13–15] and decreases of Akt phosphorylation [16] have been described upon high tidal volume ventilation in mice [13–16]. Using moderate tidal volumes with presumably lower degree of stretch in our model, we found no effect of ventilation alone on Akt phosphorylation. In contrast, H_2_S treatment clearly augmented Akt phosphorylation, both in unventilated and ventilated mice. It is important to note that in the current study Akt activity was not measured additionally; however, previous reports in other models point in the same direction: H_2_S treatment enhanced Akt phosphorylation in models of liver ischemia-reperfusion [43, 44], in lung injury due to infrarenal aortic cross-clamping [45], and bronchopulmonary dysplasia [46], suggesting a central role for Akt activation in H_2_S-mediated organ-protective effects.

In another set of animal experiments, we therefore inhibited PI3K/Akt signaling by* i.p.* application of LY294002 [12, 14]. This inhibition now affected Nox protein expression. A slight decrease in Nox2 due to H_2_S supplemented ventilation was inverted by Akt inhibition, suggesting a regulatory role for both Akt and H_2_S on Nox expression in VILI. We also found that inhibition of Akt signaling reversed a series of beneficial effects mediated by H_2_S in VILI: (1) the formation of RONS that has been reduced by H_2_S in lung tissue was reestablished, (2) the antioxidative effect of H_2_S shown as the induction of glutathione was reduced, (3) the anti-inflammatory effect of H_2_S, that is, prevention of neutrophil influx and proinflammatory cytokine release, was abolished, and (4) the organ-protective effect of H_2_S as indicated by reduced alveolar wall thickening and VILI-score was abrogated in the presence of the inhibitor. Data from few other injury models point in a similar direction. For instance, blockade of H_2_S-induced PI3K/Akt signaling restored ROS generation in aging rat hearts in ischemic-postconditioning-induced cardioprotection [47] and in a model of left ventricular remodeling in smoking rats [28]. As a consequence, infarct size was also reestablished in the latter report [28].

On a cellular level, H_2_S is known to be converted into a series of reactive sulfur species, such as disulfides, hydropersulfides, or polysulfides. All of these species are ascribed to be of biologically relevant potential [37, 48]. Polysulfides can modify cysteine residues of their target proteins by sulfuration (sulfhydration), thereby rendering their activation status [37]. From our results, we cannot conclude that Akt itself is modified directly by H_2_S or that such a modification may influence Akt phosphorylation and/or activity. Therefore, the mechanism of H_2_S-mediated Akt activation still remains elusive [49]. Transient receptor potential vanilloid type 1 (TRPV1, [50, 51]), phosphatase and tensin homolog (PTEN, [52]), and Kelch-like ECH-associated protein 1 (Keap 1, [53]) have been described to be activated due to sulfuration, by H_2_S. TRPV1, PTEN, and Keap 1 are important players in Akt activation [52, 54], also during mechanical ventilation settings [55]. It appears likely that H_2_S may also in the current study provide its protective effects by directly interfering with at least some of these proteins. With respect to GSH, H_2_S may modulate the balance of reduced (GSH) and oxidized (GSSG) glutathione by promoting the normalization of the GSSG to GSH ratio [56]. Therefore, antioxidative events may also contribute to H_2_S-mediated protection from VILI.

The present study shows for the first time that H_2_S induces Akt signaling and prevents oxidative stress in VILI. Moreover, inhibition of Akt restores not only lung injury and inflammation but also RONS production, suggesting Akt activation as one of the key events in H_2_S-induced protection from VILI.

## 5. Conclusion

Besides the inhibition of inflammatory responses, we demonstrate that H_2_S protects against VILI by limiting excessive RONS formation. We provide the first evidence that this effect is mediated via the activation of the PI3K/Akt signaling pathway. Akt activation appears to be of outstanding functional relevance for H_2_S-mediated effects. Inhibition of the PI3K/Akt pathway abolished the protective, anti-inflammatory, and antioxidative action of H_2_S during mechanical ventilation. Thus, the study exposes a new mechanistic pathway in uncovering the beneficial potential of H_2_S in the treatment of ventilator-induced lung injury.
