# Corrigendum to “Hydrogen Sulfide Prevents Formation of Reactive Oxygen Species through PI3K/Akt Signaling and Limits Ventilator-Induced Lung Injury”

**DOI:** 10.1155/2017/9230134

**Published:** 2017-10-16

**Authors:** Sashko Georgiev Spassov, Rosa Donus, Paul Mikael Ihle, Helen Engelstaedter, Alexander Hoetzel, Simone Faller

**Affiliations:** Department of Anesthesiology and Critical Care, Medical Center-University of Freiburg, Faculty of Medicine, University of Freiburg, Freiburg, Germany

In the article titled “Hydrogen Sulfide Prevents Formation of Reactive Oxygen Species through PI3K/Akt Signaling and Limits Ventilator-Induced Lung Injury” [1], there was an error in the authors' affiliation. The corrected affiliation is shown above.
